# Left Atrial Appendage Thrombus and Dense Spontaneous Echo Contrast in Anticoagulated Atrial Fibrillation Patients Referred for Cardioversion: Beyond CHA_2_DS_2_-VASc and Cardiac Implantable Electronic Devices

**DOI:** 10.3390/jcm15124500

**Published:** 2026-06-10

**Authors:** Kinga Gościńska-Bis, Michał Pieczara, Jolanta Pol-Romik, Jolanta Biernat, Magdalena Cybulska, Kamil Kempa, Eugeniusz Piłat, Tomasz Roleder, Krzysztof S. Gołba

**Affiliations:** 1 Department of Electrocardiology and Heart Failure, Upper-Silesian Medical Center, Ziolowa 45/47 Str., 40-635 Katowice, Polandkgolba@sum.edu.pl (K.S.G.); 2 Department of Electrocardiology and Heart Failure, Medical University of Silesia in Katowice, Ziolowa 45/47 Str., 40-635 Katowice, Poland; 3 Voxel S.A. Medical Diagnostic Center, Radiowa 2 Str., 44-121 Gliwice, Poland; 4 Faculty of Medicine, Wroclaw University of Science and Technology, 27 Wybrzeże Stanisława Wyspiańskiego Str., 50-370 Wrocław, Poland

**Keywords:** atrial fibrillation, left atrial appendage thrombus, spontaneous echocardiographic contrast, cardioversion, cardiac implantable electronic devices

## Abstract

**Background/Objectives:** Predictors of left atrial appendage thrombus (LAAT) in adequately anticoagulated patients with atrial fibrillation (AF) referred for direct current cardioversion (DCCV) remain insufficiently defined, particularly in populations with a high prevalence of cardiac implantable electronic devices (CIEDs). The aim of this study was to identify clinical and echocardiographic determinants of LAAT and/or dense spontaneous echocardiographic contrast (SEC) in patients with persistent AF referred for DCCV. **Methods:** This prospective observational study included 510 consecutive patients with persistent AF who had received at least 3 weeks of effective anticoagulation and underwent transthoracic and transesophageal echocardiography prior to elective DCCV. The primary endpoint was the presence of LAAT and/or dense SEC (Fatkin grade 3–4). Independent predictors were identified using multivariable logistic regression. **Results:** LAAT and/or dense SEC were detected in 192 patients (37.6%)—of whom 73 had overt LAAT, 19 had borderline LAAT, and the remainder had dense SEC (Fatkin grade 3–4). Independent predictors included lower left ventricular ejection fraction (OR 0.95 per 1% increase, 95% CI 0.94–0.97, *p* < 0.0001), reduced right ventricular fractional area change (OR 0.93 per 1% increase, 95% CI 0.91–0.94, *p* < 0.0001), larger left atrial area (OR 1.05 per 1 cm^2^ increase, 95% CI 1.01–1.09, *p* = 0.011), and female sex (OR 1.78, 95% CI 1.14–2.79, *p* = 0.012). Moderate or greater mitral regurgitation was associated with a lower risk (OR 0.50, 95% CI 0.30–0.82, *p* = 0.007). The CHA_2_DS_2_-VASc score and the presence of right ventricular leads were not independently associated with LAAT/SEC. The model showed good discrimination (AUC 0.81, 95% CI 0.77–0.84). **Conclusions:** In anticoagulated patients with persistent AF, LAAT and/or dense SEC remain common and are primarily driven by echocardiographic markers of biventricular dysfunction and left atrial remodeling rather than by traditional clinical risk scores or the presence of cardiac devices.

## 1. Introduction

Transthoracic echocardiography (TTE) and transesophageal echocardiography (TEE) remain essential components of the diagnostic evaluation of patients with atrial fibrillation (AF), both for planned therapeutic interventions and for the assessment of associated structural heart disease. According to current European Society of Cardiology (ESC) guidelines, elective direct current cardioversion (DCCV) in patients with AF lasting more than 24 h can be safely performed after a minimum of three weeks of effective anticoagulation therapy. In contrast, early cardioversion, either electrical or pharmacological, requires prior TEE to exclude the presence of intracardiac thrombus [1].

Numerous studies have investigated a wide range of echocardiographic and demographic parameters associated with an increased risk of left atrial appendage thrombus (LAAT) formation [2,3]. However, these analyses have typically included mixed populations of patients with atrial fibrillation and atrial flutter—an arrhythmia associated with a substantially lower risk of LAAT formation. Moreover, the available evidence largely derives from general AF populations with a low overall risk of thromboembolic events. There is evidence that hypercoagulability occurs in patients immediately after permanent pacemaker implantation and during the further course of the disease, particularly in those with AF [4].

The primary aim of this study was to identify which readily obtainable clinical and echocardiographic parameters are associated with the presence of either LAAT or higher degrees of spontaneous echocardiographic contrast (SEC) in a more defined patient population with a higher risk of thromboembolic events, namely, patients with atrial fibrillation referred for DCCV to a tertiary center specialized in cardiac pacing and electrophysiology, among whom a substantial proportion had previously implanted CIEDs. Given the hypercoagulability associated with cardiac device implantation, a secondary, exploratory aim was to examine whether the presence of right-heart device leads contributes independently to LAAT/SEC risk. Identification of such parameters may improve risk stratification and guide the use of TEE prior to DCCV, even in patients receiving adequate anticoagulation for over three weeks.

## 2. Methods

### 2.1. Study Design and Population

This was a prospective, observational study including patients enrolled in a dedicated registry conducted by the Department of Electrocardiology and Heart Failure at the Professor Leszek Giec Upper Silesian Medical Center, Medical University of Silesia in Katowice, a tertiary referral center specialized in arrhythmia treatment and cardiac device implantation, from January 2018 to December 2022. The study population consisted of consecutive adult patients (aged ≥ 18 years) with persistent AF, confirmed on a 12-lead ECG, with a duration of 3 weeks to 12 months, referred for elective DCCV. All patients had received effective anticoagulation for at least 3 weeks prior to DCCV, either with direct oral anticoagulants (DOACs) or with therapeutic doses of vitamin K antagonists (VKAs). The quality of anticoagulation was systematically verified before enrollment. In patients receiving VKA therapy, a minimum of two INR measurements obtained during the month preceding the study were required, together with an additional INR drawn on the morning of the examination. Only patients in whom all available INR values were >2.0 were considered to have effective VKA anticoagulation and were included. In patients receiving DOACs, uninterrupted daily intake during the three weeks preceding the study was verified by structured interview. Any self-reported interruption of treatment lasting ≥ 24 h during this period led to exclusion from the analysis. Exclusion criteria included atrial flutter or tachycardia, a history of mechanical prosthetic valve presence, subtherapeutic anticoagulation (INR < 2.0 for VKA, non-adherence to DOAC therapy as defined above), previous left atrial appendage occlusion, a poor TTE window or contraindications to TEE. In addition to echocardiographic parameters, the registry collected clinical data, comorbidities, pharmacotherapy, and the type of anticoagulant treatment.

### 2.2. Echocardiographic Protocol

Each patient underwent a comprehensive transthoracic echocardiogram (TTE) followed by a transesophageal echocardiogram (TEE) within 24 h of the planned DCCV. All examinations were performed within the Echocardiography Laboratory of the Department of Electrocardiology and Heart Failure. The examinations were carried out by a team of four cardiologists certified in echocardiography using a Philips iE33 or Philips Epiq 7 (Philips Medical Systems, Andover, MA, USA). TTE and TEE examinations were performed in accordance with the current recommendations of the European Association of Cardiovascular Imaging (EACVI) and the American Society of Echocardiography (ASE) for adult echocardiography [5]. Echocardiographers had clinical access to basic patient information required for safe TEE performance (anticoagulation status, comorbidities, presence of cardiac implantable electronic devices) but were not separately blinded to these data; formal blinded re-reading of LAAT/SEC findings was not performed.

During TEE, the left atrial appendage (LAA) was systematically imaged from multiple standard transducer angles, including X-plane views, to verify the presence of thrombus or SEC. SEC was defined as swirling echo density within the left atrium or LAA despite appropriate gain settings and was graded on a scale from 0 to 4 according to the classification originally proposed by Fatkin [6,7]. Patients with SEC of grade 3 or 4, corresponding to moderate to dense swirling echodensity evident throughout the cardiac cycle, were considered to have an equivalent risk profile to those with LAAT. Borderline LAAT was defined as a discrete echodensity within the left atrial appendage that could not be unequivocally classified as a fully formed thrombus; such cases were adjudicated by two independent experts and included within the composite LAAT/SEC endpoint.

During transthoracic echocardiography, the following parameters were assessed: left ventricular ejection fraction (LVEF); left atrial (LA) area; right atrial (RA) area; tricuspid annular plane systolic excursion (TAPSE); right ventricular fractional area change (FAC); and the severity of valvular heart disease was graded as none, mild, moderate, or severe and assessed separately for each of the following: tricuspid regurgitation (TR); mitral regurgitation (MR) and mitral stenosis; and aortic regurgitation and aortic stenosis.

In accordance with Resolution No. KNW/0022/KB/15/18 dated 30 January 2018, the Bioethics Committee of the Medical University of Silesia in Katowice determined that the study was purely observational and exempt from formal bioethical approval.

### 2.3. Statistical Analysis

Continuous variables were expressed as mean ± standard deviation (SD), and categorical variables as absolute numbers and percentages. Comparisons between two independent groups were performed using the unpaired Student’s *t*-test for continuous variables and the chi-square test for categorical variables, as appropriate. The distribution of continuous variables was examined before analysis using histograms and the Shapiro–Wilk test. Given the large sample size (n = 510), the unpaired *t*-test was retained for comparisons of means because it is robust to departures from normality in large samples through the central limit theorem, rather than because the variables were themselves normally distributed [8]. The demographic variables, echocardiographic data, clinical information, comorbidities, and pharmacotherapy options listed in Table 1 are first assessed using univariate analysis to evaluate their association with LAAT and/or SEC. Variables with *p*-values of 0.1 or lower in the univariate analysis were subsequently entered into a stepwise multivariable logistic regression model to identify independent predictors of LAAT and/or SEC. Variables with pronounced right-skew (body mass index and left and right atrial area) were instead summarized as median [interquartile range] and compared between groups with the Mann–Whitney U test, as indicated in Table 1. In addition, all between-group comparisons were repeated using the Mann–Whitney U test as a sensitivity analysis; conclusions were concordant for all variables retained in the model. With 192 events and five predictors in the final model, the number of events per variable (approximately 38) substantially exceeded the conventional minimum of 10, making severe overfitting unlikely a priori. For continuous independent predictors, the cut-off value was determined for each using receiver operating characteristic (ROC) curve analysis. Model performance was assessed using ROC curve analysis, and discriminative ability was quantified by the area under the curve (AUC). Reporting followed the Transparent Reporting of a Multivariable Prediction Model for Individual Prognosis or Diagnosis (TRIPOD) statement [9]. In addition to discrimination, calibration was assessed using the Hosmer–Lemeshow goodness-of-fit test, a calibration plot of observed versus predicted probabilities, the calibration intercept and slope, and the Brier score. To quantify and correct for optimism, the model was internally validated by bootstrap resampling (1000 resamples), yielding an optimism-corrected AUC and calibration slope. Risk-of-bias considerations were additionally informed by the Prediction Model Risk Of Bias Assessment Tool (PROBAST) framework, encompassing the participant, predictor, outcome, and analysis domains [10]. A two-sided *p*-value < 0.05 was considered statistically significant for all analyses. Statistical analyses were performed using MedCalc Statistical Software, version 23.4.9 (MedCalc Software Ltd., Ostend, Belgium); calibration metrics and bootstrap internal validation were computed in Python 3 (scikit-learn and SciPy).

**Table 1:** Baseline demographic, clinical, and echocardiographic characteristics according to the presence of left atrial appendage thrombus and/or spontaneous echocardiographic contrast (LAAT/SEC).

Variable	Total N	LAAT/SEC Detected	LAAT/SEC Not Detected	*p*-Value
Patients n (%)	510	192 (37.6)	318 (62.4)	-
Age (years)	510	68.2 (9.4)	70.3 (9.5)	0.0121
Body mass index (kg/m^2^)	510	29.7 [27.5–32.7]	29.4 [27.0–32.8]	0.613
Sex: female n (%)	193	76 (39.6)	117 (60.6)	0.0032
Left ventricle ejection fraction (LVEF, %)	510	44.3 ± 14.3	52.7 ± 11.7	<0.001
Right ventricle fractional area change (FAC, %)	510	29.7 ± 12.0	40.8 ± 12.2	<0.001
Tricuspid annular plane systolic excursion (TAPSE, mm)	510	18.1 ± 4.8	19.7 ± 4.2	0.0002
Left atrial area (cm^2^)	510	26.0 [23.0–30.0]	24.1 [21.0–27.5]	0.0001
Right atrial area (cm^2^)	510	24.0 [21.0–29.6]	21.8 [18.8–25.3]	<0.0001
Right ventricle lead present n (%)	300	121 (63.0)	179 (56.3)	0.1349
Coronary artery disease n (%)	283	105 (37.1)	170 (62.9)	0.7771
Diabetes mellitus n (%)	129	54 (41.9)	75 (58.1)	0.2536
Hypertension n (%)	397	147 (37.0)	250 (63.0)	0.5888
Estimated glomerular filtration rate * eGFR (mL/min/1.73 m^2^) *	510	53.5 (9.8)	51.6 (12.5)	0.0610
Previous ischemic stroke or TIA n (%)	33	11 (33.3)	22 (66.7)	0.5973
Previous myocardial infarction n (%)	86	38 (44.2)	48 (55.8)	0.1703
Tricuspid regurgitation ≥ moderate n (%)	192	91 (47.4)	101 (30.5)	<0.0001
Mitral regurgitation ≥ moderate n (%)	125	39 (20.3)	86 (27.0)	0.0872
Mitral stenosis ≥ moderate n (%)	9	3 (1.6)	6 (1.9)	0.7878
Aortic regurgitation ≥ moderate n (%)	37	12 (6.2)	25 (7.9)	0.4971
Aortic stenosis ≥ moderate n (%)	11	3 (1.6)	8 (2.5)	0.4732
CHA_2_DS_2_-VASc score **	510	192 (37.6)	318 (62.4)	0.3393
Anticoagulation therapy: DOAC n (%)	275	92 (33.5)	183 (66.5)	<0.0001
Anticoagulation therapy: Vitamin K Antagonists n (%)	235	100 (42.6)	135 (57.4)	0.0224
Time of atrial fibrillation onset to TEE (months)	510	6.8 (7.1)	6.2 (6.9)	0.3030

**Figure 1:**
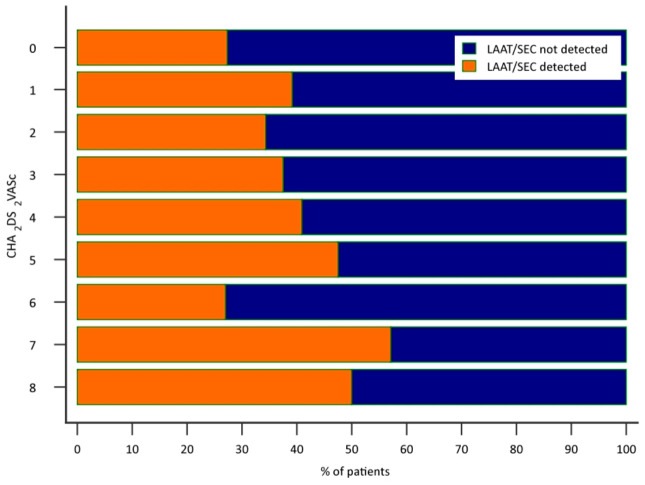
Left atrial appendage thrombus and/or spontaneous echocardiographic contrast detection distribution at different CHA_2_DS_2_-VASc score levels.

LAAT/SEC—according to the Fatkin SEC Classification System [6]. LVEF—left ventricular ejection fraction, FAC—fractional area change, TAPSE—tricuspid annular plane systolic excursion, eGFR—estimated glomerular filtration rate, DOAC—Direct Oral Anticoagulants. TIA—transient ischemic attack. TEE—transesophageal echocardiography. (*)—using the Cockcroft–Gault equation. (**)—LAAT/SEC detection distribution at different CHA_2_DS_2_-VASc score levels; see Figure 1. Continuous variables are presented as mean ± SD and compared with the unpaired Student’s *t*-test, except markedly right-skewed variables (body mass index, left atrial area, and right atrial area), which are presented as median [interquartile range] and compared with the Mann–Whitney U test.

### 2.4. Use of Artificial Intelligence

Artificial intelligence (AI)-assisted tools were not used in the collection, analysis, or interpretation of study data. AI language model assistance (Claude, Anthropic, San Francisco, CA, USA) was used solely for linguistic revision and manuscript editing purposes. The authors retain full responsibility for the integrity, accuracy, and originality of the reported research and the final content of the manuscript.

## 3. Results

### 3.1. Study Population

Between January 2018 and December 2022, transesophageal echocardiography (TEE) was performed in 685 consecutive patients prior to scheduled DCCV. Of these, 167 patients were excluded because of atrial tachycardia or atrial flutter on electrocardiography (ECG), and an additional 8 patients were excluded for other predefined exclusion criteria. Ultimately, 510 patients with AF (without concomitant atrial flutter or atrial tachycardia) were included in the final analysis. Baseline demographic characteristics, cardiac function and morphology, comorbidities, and medical history of the study population are summarized in Table 1.

### 3.2. Univariate Analysis

Among the 510 included patients, left atrial appendage thrombus and/or dense spontaneous echocardiographic contrast (LAAT/SEC) was detected in 192 individuals (37.6%). Of these 192 patients, 73 (38.0%) had overt LAAT, 19 (9.9%) had borderline LAAT, and the remaining 100 (52.1%) had dense SEC (Fatkin grade 3 or 4) as the index finding. Compared with patients without LAAT/SEC, those with LAAT/SEC demonstrated significantly impaired biventricular function, with lower left ventricular ejection fraction (44.3 ± 14.3% vs. 52.7 ± 11.7%, *p* < 0.001), reduced right ventricular fractional area change (29.7 ± 12.0% vs. 40.8 ± 12.2%, *p* < 0.001), and lower tricuspid annular plane systolic excursion (18.1 ± 4.8 mm vs. 19.7 ± 4.2 mm, *p* = 0.0002). In addition, both left and right atrial areas were significantly larger in the LAAT/SEC group (*p* ≤ 0.0001 for both).

Moderate or greater tricuspid regurgitation (TR) was more frequently observed in patients with LAAT/SEC (47.4% vs. 30.5%, *p* < 0.0001); however, it did not retain independent significance in the multivariable model. Patients with LAAT/SEC were younger than those without LAAT/SEC (68.2 ± 9.4 vs. 70.3 ± 9.5 years, *p* = 0.012), and the proportion of women was higher in the LAAT/SEC group (39.6% vs. 36.6% in males). No significant differences were observed in body mass index, comorbidities including coronary artery disease, diabetes mellitus, hypertension, renal function, and prior thromboembolic events, or atrial fibrillation duration. The CHA_2_DS_2_-VASc score was not associated with LAAT/SEC presence. Moreover, both groups, with and without LAAT/SEC, showed a similar distribution across levels of the CHA_2_DS_2_-VASc score; the *p* for trend was 0.53 (Figure 1). However, treatment with DOACs was associated with a lower relative risk of LAAT/SEC compared with VKAs (RR 0.86; 95% CI 0.75–0.97; *p* = 0.04).

### 3.3. Multivariable Logistic Regression Analysis

Variables significantly associated with LAAT/SEC in univariate analysis were entered into a stepwise multivariable logistic regression model. The final model demonstrated good overall fit (χ^2^ = 146.0, *p* < 0.0001; Nagelkerke R^2^ = 0.34) and acceptable discriminative ability (AUC = 0.81, 95% CI 0.77–0.84). Independent predictors of LAAT/SEC were lower left ventricular ejection fraction (OR 0.95 per 1% increase, 95% CI 0.94–0.97, *p* < 0.0001), reduced right ventricular fractional area change (OR 0.93 per 1% increase, 95% CI 0.91–0.94, *p* < 0.0001), female sex (OR 1.78, 95% CI 1.14–2.79, *p* = 0.012), and larger left atrial area (OR 1.05 per 1 cm^2^ increase, 95% CI 1.01–1.09, *p* = 0.011). In contrast, the presence of at least moderate MR was independently associated with a lower likelihood of LAAT/SEC detection (OR 0.50, 95% CI 0.30–0.82, *p* = 0.007).

**Figure 2:**
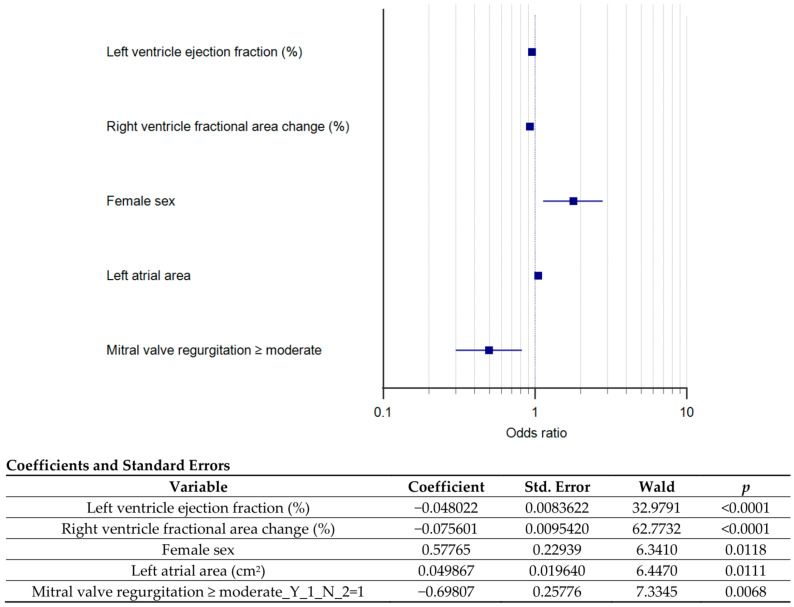
Prediction model for the occurrence of left atrial appendage thrombus and/or spontaneous echo contrast on transesophageal echocardiography.

**Figure 3:**
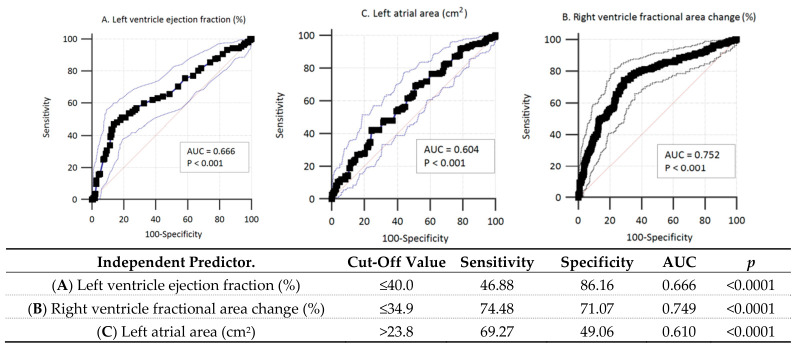
Cut-off values for continuous independent predictor variables with corresponding receiver operating characteristic curve analysis.

The multivariable model correctly classified 75.9% of cases, with a sensitivity of 61.1% and specificity of 84.9% for LAAT/SEC detection. Independent predictors and their corresponding odds ratios are illustrated in the forest plot (Figure 2). Cut-off values for continuous independent predictors, along with corresponding data from receiver operating characteristic curve analysis, are shown in Figure 3.

**Figure 4:**
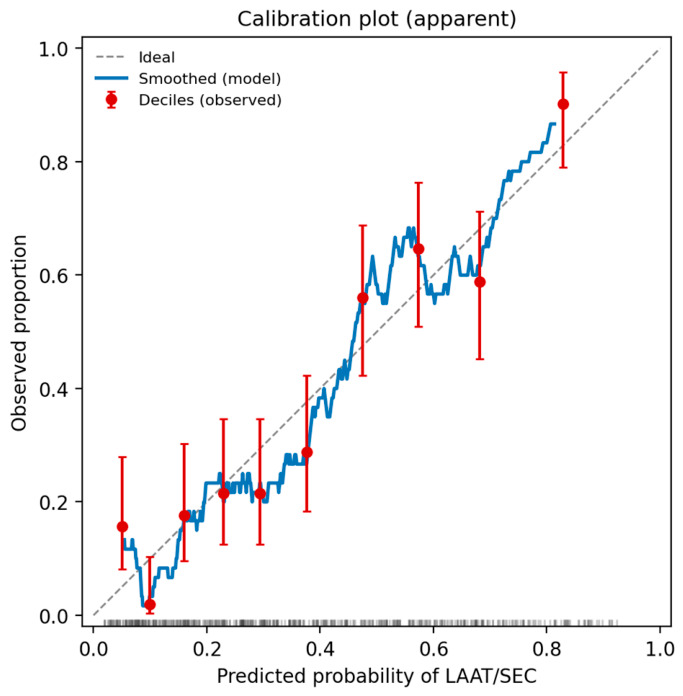
Calibration plot of the multivariable model (apparent). Red points are observed event proportions by decile of predicted risk with 95% confidence intervals; the blue line is a smoothed estimate; the dashed line denotes perfect calibration. Tick marks along the horizontal axis show the distribution of predicted probabilities.

Beyond discrimination (apparent AUC 0.81, 95% CI 0.77–0.84), calibration was assessed in detail. Calibration-in-the-large was satisfactory: the mean predicted probability equaled the observed event rate (0.376), with a calibration intercept of 0.00 and an apparent calibration slope of 1.00. The Brier score was 0.17. The Hosmer–Lemeshow test indicated some departure from perfect calibration within risk strata (χ^2^ = 25.0, df = 8, *p* = 0.002 with 10 groups), although this result was sensitive to the number of groups (*p* = 0.10 with 8 groups), in keeping with the known instability of this test; the calibration plot (Figure 4) showed that observed and predicted probabilities tracked the line of identity across most of the range, with wider scatter confined to the lowest-risk deciles. On internal validation by bootstrapping (1000 resamples), the mean optimism in the AUC was only 0.007, giving an optimism-corrected AUC of 0.80, and the optimism-corrected calibration slope was 0.96, indicating minimal overfitting and only modest shrinkage of the predictor effects.

## 4. Discussion

The present study identified several readily available echocardiographic and clinical parameters that were independently associated with LAAT/SEC presence in patients with AF referred for DCCV. In the present cohort of 192 patients with LAAT/SEC, 73 (38.0%) had a clearly delineated thrombus, 19 (9.9%) presented with borderline findings, and 100 (52.1%) exhibited dense SEC (Fatkin grade 3–4) as the index finding—underscoring that the majority of detected prothrombotic features were ‘pre-thrombotic’ rather than fully formed thrombi. Importantly, the proportion of patients with LAAT/SEC in our cohort was higher than reported previously [9,10]. This finding is largely explained by two key methodological aspects: the inclusion of dense SEC (Fatkin grades 3–4) as a thrombus-equivalent and the exclusion of patients with atrial flutter, an arrhythmia associated with substantially lower thromboembolic risk and often included in earlier studies.

### 4.1. Inclusion of Spontaneous Echocardiographic Contrast and Exclusion of Atrial Flutter as Key Methodological Distinctions

SEC represents a recognized marker of blood stasis and hypercoagulability within the LA and LA appendage and has been consistently associated with an increased risk of thromboembolic events [6,7,11,12]. By incorporating dense SEC (Fatkin grades 3–4) into the composite endpoint, our analysis captures an earlier and clinically relevant stage of thrombogenesis rather than limiting the assessment to fully formed thrombi. This approach provides a more sensitive reflection of the prothrombotic milieu. Considering higher grades of SEC as part of the thromboembolic risk assessment—rather than restricting evaluation to overt thrombus—broadens the spectrum of detectable LA appendage pathology and may improve the sensitivity of stroke risk prediction. In a retrospective observational cohort by Turek et al. that included 500 patients with AF on chronic oral anticoagulation followed over 13 years, LA appendage abnormalities—most commonly SEC—were identified in roughly one-fifth of patients undergoing TEE and were more frequent in those with prior stroke, diabetes, heart failure, or vascular disease; SEC, even without overt thrombus, was independently associated with thromboembolic events and all-cause mortality [13]. Similarly, the observational cohort underpinning the CLOTS-AF score by Segan et al. confirmed high rates of LAAT/SEC at TEE before DCCV, supporting the relevance of dense SEC as part of the composite endpoint [14]. The LAAT/SEC approach likely explains the higher detection rate observed in our cohort. In summary, it is worth considering the permanent inclusion of dense SEC in the assessment of stroke risk in patients with AF, either before DCCV or before pulmonary vein isolation ablation.

Furthermore, unlike many prior studies, we deliberately excluded patients with atrial flutter. This approach avoids analyzing a mixed population containing both AF and atrial flutter, as the latter’s presence may dilute associations with LAAT/SEC because of its lower thromboembolic risk [15,16]. Restricting the analysis exclusively to AF-only patients yielded a more homogeneous population and enhanced the ability to identify true predictors of LAAT/SEC in this population. This methodological distinction should not, however, be overstated: atrial flutter and atrial fibrillation frequently coexist in clinical practice and may share overlapping structural and electrical substrates, so the difference in LAAT prevalence between the two arrhythmias represents a gradient rather than a categorical dichotomy.

### 4.2. Left Ventricular Ejection Fraction and Left Atrial Size

Reduced left ventricular ejection fraction and left atrial enlargement were independent predictors of LAAT/SEC in our study. These findings are consistent with well-established pathophysiological mechanisms and, therefore, not unexpected. Impaired left ventricular systolic function promotes blood stasis within the left atrium through elevated filling pressures, reduced forward flow, and adverse hemodynamic conditions, all of which favor thrombus formation. Similarly, left atrial enlargement reflects chronic pressure and volume overload as well as advanced structural remodeling, including fibrosis and loss of contractile function, which further contribute to impaired atrial emptying and local thrombogenesis. Among meta-analyses, Froehlich et al. pooled data from cohort studies of patients with and without AF and demonstrated that larger LA dimensions were independently associated with higher rates of stroke and cardiovascular events, supporting LA size as a robust surrogate of thromboembolic substrate [17]. More broadly, these structural changes can be viewed within the framework of atrial cardiomyopathy, in which progressive structural, electrical, and mechanical remodeling of the atrial myocardium contributes to thromboembolic risk independently of rhythm status and can be characterized by multimodal imaging, including the left atrial volume index [18,19]. Among observational studies, Cemri et al. showed that depressed LVEF in chronic non-valvular AF was accompanied by reduced LA appendage emptying velocities and impaired LA mechanical function [20]; Handke et al. identified reduced LVEF as the strongest predictor of LA SEC and thrombus among stroke patients in sinus rhythm [21]; and Black et al. provided an early observational synthesis linking LA SEC to a cardiogenic source of embolism [22]. Our findings reinforce the concept that echocardiographic markers of cardiac dysfunction and atrial remodeling remain central to thromboembolic risk, even in adequately anticoagulated patients, and may provide incremental value beyond clinical risk scores in identifying individuals at higher risk of LAAT prior to cardioversion.

More broadly, LAAT and dense SEC may be regarded as the visible echocardiographic expression of an underlying atrial cardiomyopathy rather than as appendage-localized phenomena. According to the EHRA/HRS/APHRS/SOLAECE expert consensus, atrial cardiomyopathy encompasses structural, architectural, contractile, and electrophysiological changes in the atrial myocardium with the potential to produce clinically relevant manifestations—including arrhythmia and thromboembolism—irrespective of the presence of AF [23]. Within this framework, the biventricular dysfunction and left atrial enlargement observed in our cohort can be understood as integrated markers of a diseased atrial substrate, which clinical risk scores may not fully capture. This perspective also provides a rationale for incorporating imaging-based substrate assessment alongside, rather than in place of, validated clinical scores when stratifying thromboembolic risk in persistent AF.

### 4.3. Right Ventricular Dysfunction and TAPSE

Right ventricular systolic dysfunction emerged as an important determinant of LAAT/SEC risk in our study. Importantly, right ventricular fractional area change (FAC) remained an independent predictor in the multivariable model, underscoring the relevance of global right ventricular systolic performance in the pathophysiology of left atrial thrombogenesis. Reduced FAC reflects impaired right ventricular contractility and is associated with elevated right-sided pressures, ventricular interdependence, and compromised left ventricular filling, all of which may contribute to left atrial blood stasis and thrombus formation. Although TAPSE did not remain an independent predictor in the multivariable model, its strong association in univariate analysis aligns with emerging evidence highlighting the role of right ventricular function in left atrial thrombogenesis.

Notably, TAPSE < 17 mm constitutes a major component of the recently proposed CLOTS-AF score [20], contributing 2 points to risk stratification and representing the largest attempt to incorporate right ventricular functional parameters into the prediction of LAAT. In the observational derivation cohort underpinning the CLOTS-AF score, anticoagulated AF patients with LAAT had significantly lower TAPSE values than those without thrombus, and TAPSE < 17 mm was retained as an independent predictor of LAAT in multivariable modeling [14]. These findings support the concept that impaired right ventricular longitudinal function reflects advanced cardiac disease and adverse hemodynamic conditions favoring atrial blood stasis. Our findings extend this concept by demonstrating that FAC, as a more comprehensive measure of right ventricular systolic function, may provide additional and independent prognostic value in identifying patients at increased risk of LAAT/SEC.

### 4.4. Tricuspid Regurgitation and Right Heart–Left Heart Interaction

TR, particularly when moderate or severe, often accompanies right ventricular dysfunction and is an integral part of the pathophysiological cascade leading to thrombus formation. TR results in elevated right atrial pressures, altered ventricular interdependence, and impaired left-heart filling dynamics. In patients with atrial fibrillation, these hemodynamic disturbances may exacerbate left atrial stasis and promote thrombus formation within the left atrial appendage [24].

In our cohort, moderate or greater TR was significantly more frequent among patients with LAAT/SEC on univariate analysis but lost independent significance in the multivariable model. This is most plausibly explained by close collinearity with right ventricular fractional area change, which captures the underlying right-heart dysfunction more directly and remained an independent predictor of LAAT/SEC.

In a single-center observational cohort of patients with isolated severe TR and AF, Ogawa et al. demonstrated that impaired right ventricular free-wall longitudinal strain was independently associated with all-cause mortality, supporting the prognostic relevance of right-heart functional remodeling in this population [25]. Our findings further support the notion that right-sided valvular disease and ventricular dysfunction should not be overlooked when assessing thromboembolic risk in atrial fibrillation.

### 4.5. Mitral Regurgitation

Interestingly, moderate or greater MR was associated with a lower likelihood of LAAT/SEC in our cohort. This observation is consistent with prior reports suggesting a potential “washout” effect of significant MR, whereby increased left atrial flow and turbulence may reduce blood stasis in the left atrial appendage. In a large observational study by Melduni et al. of patients with non-valvular AF undergoing TEE, the presence of at least moderate MR was independently associated with a significantly lower prevalence of LA appendage thrombus and a lower risk of stroke and systemic embolism, supporting the “protective” hemodynamic effect of MR on LA appendage stasis [26]. However, available evidence on this relationship remains inconsistent, and MR is also a marker of advanced structural heart disease, which may confound its association with thrombus formation. Therefore, this finding should be interpreted with caution and considered hypothesis-generating rather than definitive.

### 4.6. Atrial Remodeling, Pacing, and Intracardiac Leads

Structural remodeling of the left atrium represents a central mechanism underlying LAAT/SEC formation. In a retrospective single-center analysis of 461 patients undergoing right ventricular pacing reported by Lee et al., long-term right ventricular pacing was associated with the development of left atrial enlargement and pacing-induced deterioration of left ventricular function, with a higher pacing burden identified as a major risk factor [27]. Together, these changes create conditions that favor blood stasis within the left atrial appendage. In addition, the presence of intracardiac leads has been linked to a prothrombotic local environment, with lead-associated thrombus identified in a clinically relevant minority of device recipients and occasional case reports of thromboembolic complications [28,29,30].

In the present study, however, the presence of a right ventricular lead was not independently associated with LAAT or dense SEC. This null finding should be interpreted as the main message of this subsection: any local procoagulant effect related to right-heart leads appears to be largely outweighed by the dominant influence of underlying cardiac dysfunction and left atrial remodeling, which remain the principal determinants of thrombogenesis in this population. It is worth noting that current risk stratification tools, including the CLOTS-AF score [14], do not account for the presence of cardiac implantable electronic device leads, and our data do not support adding this variable to such models.

### 4.7. Age Paradox and Sex-Related Differences in LAAT/SEC Risk

An intriguing finding of the present study was that patients with LAAT/SEC were significantly younger than those without (68.2 ± 9.4 vs. 70.3 ± 9.5 years, *p* = 0.012), despite female sex emerging as an independent predictor of LAAT/SEC (OR 1.78, 95% CI 1.14–2.79). This apparent paradox is most plausibly explained by the higher proportion of women in the LAAT/SEC group. In AF, female sex has been associated—independently of age—with a more pronounced left atrial structural substrate, including greater atrial fibrosis, more pronounced diastolic dysfunction, and sex-specific differences in hemostatic balance [31]. We interpret our finding as an observational association rather than as evidence of a distinct aetiopathogenic mechanism, and the underlying biological pathways remain to be established. Importantly, the CLOTS-AF score similarly did not identify female sex or age as independent predictors of LAAT [14], suggesting that age-based clinical risk scores may not fully capture the structural substrate driving left atrial thrombogenesis. In a multicenter observational analysis by Ciba-Stemplewska et al. of patients with AF treated with dabigatran or rivaroxaban, LAAT predictors differed between men and women; non-paroxysmal AF was the dominant predictor in women, whereas other clinical and echocardiographic features prevailed in men, highlighting the heterogeneity of thrombogenic mechanisms by sex [32]. Our findings support the hypothesis that sex-related differences in atrial remodeling contribute independently to left atrial thrombogenesis and should be incorporated into future imaging-based risk stratification models.

### 4.8. The CHA_2_DS_2_-VASc Score

The CHA_2_DS_2_-VASc score was not significantly associated with LAAT/SEC in our cohort (odds ratio per 1-point increase 1.07, 95% CI 0.94–1.21; *p* = 0.34), which should not be interpreted as a limitation of the score itself for its intended use. The CHA_2_DS_2_-VASc score was developed and validated for estimating long-term clinical thromboembolism (stroke and systemic embolism) in non-valvular AF, not for identifying patients with an imaging-detected atrial thrombogenic substrate on a single cross-sectional TEE assessment. Our finding therefore reflects a known structural limitation of clinical risk scores in this specific imaging context: they do not incorporate direct markers of left atrial structural remodeling, blood stasis, or appendage function [33].

Among meta-analyses, Sun et al. pooled data from observational studies in non-valvular AF and demonstrated that although higher CHA_2_DS_2_-VASc scores were associated with greater odds of LAAT/SEC, the score’s discriminative ability for imaging-detected thrombus was only modest [34]. Among observational studies, Michalska et al., in a multicenter analysis of AF patients treated with non-vitamin K antagonist oral anticoagulants, similarly reported that the CHA_2_DS_2_-VASc score performed only modestly for predicting LAAT on TEE [35]. These observations highlight the importance of complementary echocardiographic assessment in selected patients.

### 4.9. Anticoagulant Protection with DOACs vs. VKAs—Confirmed Benefit in a High-Risk Cohort

It is believed that DOACs provide significantly better protection against LAAT/SEC formation than VKAs in patients with any form of non-valvular AF. In our registry of patients with persistent AF, a substantial proportion of whom had intracardiac pacemaker leads, treatment with DOACs was associated with a significantly lower relative risk of LAAT/SEC compared with VKAs (RR 0.86; 95% CI 0.75–0.97; *p* = 0.04). This finding is consistent with previously published evidence, while offering a more conservative estimate of the magnitude of benefit, and extends the available data to a clinically distinct and underrepresented population.

Among meta-analyses, Cheng et al. pooled 13 studies (n = 8609 patients with AF) and demonstrated a significantly lower incidence of LAAT under DOAC therapy compared with VKA (pooled RR 0.65; 95% CI 0.47–0.90; *p* = 0.009), as well as a greater probability of thrombus resolution with DOACs (pooled OR 1.52; 95% CI 1.02–2.26; *p* = 0.040) [36]. Our RR of 0.86, while statistically significant, reflects a more attenuated risk reduction—a 14% relative decrease compared with the 35% reduction reported in that meta-analysis. This discrepancy is likely attributable to differences in study population characteristics rather than a true divergence in biological effect.

Not all observational studies, however, have demonstrated differences between DOACs and VKAs for this endpoint. In a prospective observational study by Gawałko et al. of 859 AF patients undergoing TEE before DCCV or ablation, the incidence of LA appendage thrombus (6.9% vs. 5.5%; *p* = 0.40) and dense SEC (5.3% vs. 3.3%; *p* = 0.18) was numerically lower in DOAC-treated patients compared with those receiving VKAs but did not reach statistical significance [37]. Similarly, Karwowski et al., in a single-center observational study of 160 AF patients undergoing TEE before DCCV, observed comparable rates of LAAT (8.9% vs. 3.6%) and SEC (12.4% vs. 18.5%) between DOAC and VKA groups, without statistically significant differences [38]. The failure to detect differences in both cited studies may reflect the inclusion of heterogeneous AF subtypes, paroxysmal AF forms with known lower baseline thrombogenic activity, and variable SEC grading criteria.

Our cohort consisted exclusively of patients with persistent AF, characterized by progressive structural atrial remodeling, impaired LA appendage contractile function, and a higher baseline prothrombotic burden, all of which may reduce the relative benefit conferred by any anticoagulant class compared with populations including paroxysmal AF [12,37]. Additionally, our data, which used a prospective registry design and a standardized composite endpoint encompassing both thrombus and dense SEC, may yield higher statistical sensitivity than these earlier reports. It should be emphasized, however, that the present study was not designed to compare anticoagulant efficacy and that the observed difference between DOAC- and VKA-treated patients should be interpreted with caution. As discussed in the Limitations, residual confounding from therapeutic selection—such as preferential prescription of DOACs in patients with better renal function or fewer drug interactions—cannot be excluded, and the present finding should be regarded as hypothesis-generating rather than evidence of superior anticoagulant efficacy.

### 4.10. Clinical Implications

Our findings emphasize that echocardiographic markers of global cardiac dysfunction, including biventricular systolic impairment and atrial enlargement, provide clinically relevant information beyond traditional clinical risk scores. Identification of such features may help refine patient selection for pre-procedural transesophageal echocardiography, even in individuals who have received guideline-recommended anticoagulation for more than three weeks.

## 5. Limitations and Conclusions

This study has several limitations. First, although the study is a prospective registry, it is still a single-site study conducted at a tertiary electrophysiology referral center, which may limit generalizability. Second, although all patients received at least 3 weeks of effective anticoagulation and quality of anticoagulation was systematically verified by both serial INR monitoring (for VKAs) and structured interviews regarding daily intake (for DOACs), anticoagulation quality was not directly assessed in terms of time in therapeutic range for vitamin K antagonists or formal pharmacokinetic adherence measurements for direct oral anticoagulants. Furthermore, the present study was not designed as a head-to-head comparison of anticoagulant classes, and a formal comparison of baseline clinical and echocardiographic characteristics between patients receiving DOACs and those receiving VKAs was not performed; the observed difference in LAAT/SEC prevalence between the two groups should therefore be interpreted with caution and may reflect, at least in part, residual confounding from therapeutic selection. Third, advanced echocardiographic parameters such as left atrial strain, left atrial appendage flow velocities, and right ventricular speckle-tracking strain were not routinely evaluated. Fourth, the primary endpoint combined LAAT and dense SEC (Fatkin grade 3–4) into a single composite, which, although clinically justifiable as both reflect a prothrombotic milieu, merges two distinct pathophysiological entities; this methodological choice influences the observed prevalence and limits direct comparison with cohorts that restricted the endpoint to overt thrombus. Fifth, although all examinations were performed by a small team of four certified echocardiographers using a standardized protocol, formal inter-observer reproducibility of SEC grading was not assessed; given that SEC grading remains a semi-quantitative visual assessment, residual variability cannot be entirely excluded. Finally, long-term clinical outcomes after cardioversion were not assessed.

Nevertheless, the large sample size, prospective design, homogeneous population restricted exclusively to persistent AF, and comprehensive echocardiographic protocol constitute important strengths supporting the validity of our findings. It should also be noted that although pacemaker lead presence did not emerge as an independent predictor of LAAT/SEC in multivariable analysis, residual confounding from unmeasured device-related variables—such as lead position, pacing burden, or right ventricular pacing percentage—cannot be fully excluded. A further methodological caveat concerns the modeling approach: the model was derived using univariable screening followed by stepwise selection, which may favor selection instability and optimistic performance estimates. Although bootstrap internal validation indicated only minimal optimism (optimism-corrected AUC 0.80; calibration slope 0.96), and the Hosmer–Lemeshow test suggested imperfect calibration confined to the lowest-risk strata, these estimates were obtained in the development cohort only. External validation in an independent population is therefore required before the model is used to guide selection for transesophageal echocardiography, and the proposed model should be regarded as hypothesis-generating rather than as a finalized, ready-to-deploy clinical tool.

In conclusion, the high prevalence of LAAT/SEC observed in this study reflects deliberate methodological choices aimed at capturing clinically meaningful thrombotic risk. Both right and left ventricular dysfunction and left atrial remodeling appear to play significant roles in the increased risk of left atrial appendage thrombogenesis, alongside a hypothesis-generating inverse association between moderate or greater mitral regurgitation and LAAT/SEC, which requires confirmation in independent cohorts. These factors should be incorporated into future risk-stratification strategies for patients with AF.

## Data Availability

The raw data supporting the conclusions of this article will be made available by the authors on request.
